# In Vitro Cytotoxicity Assessment of an Orthodontic Composite Containing Titanium-dioxide Nano-particles

**DOI:** 10.5681/joddd.2013.031

**Published:** 2013-12-18

**Authors:** Farzin Heravi, Mohammad Ramezani, Maryam Poosti, Mohsen Hosseini, Arezoo Shajiei, Farzaneh Ahrari

**Affiliations:** ^1^Dental Research Center, School of Dentistry, Mashhad University of Medical Sciences, Mashhad, Iran; ^2^Pharmaceutical and Biotechnology Research Center, School of Pharmacy, Mashhad University of Medical Sciences, Mashhad, Iran; ^3^Assistant Professor, Department of Orthodontics, School of Dentistry, Islamic Azad University, Tehran, Iran; ^4^School of Dentistry, Mashhad University of Medical Sciences, Mashhad, Iran

**Keywords:** Adhesive, biocompatibility, cytotoxicity, nano-particles, orthodontics, titanium dioxide

## Abstract

***Background and aims.*** Incorporation of nano-particles to orthodontic bonding systems has been considered to prevent enamel demineralization around appliances. This study investigated cytotoxicity of Transbond XT adhesive containing 1 wt% titanium dioxide (TiO_2_) nano-particles.

***Materials and methods.*** Ten composite disks were prepared from each of the conventional and TiO_2_-containg composites and aged for 1, 3, 5, 7 and 14 days in Dulbecco’s Modified Eagle’s Medium (DMEM). The extracts were obtained and exposed to culture media of human gingival fibroblasts (HGF) and mouse L929 fibroblasts. Cell viability was measured using the 3-(4,5-dimethylthiazol-2-yl)-2,5-diphenyltetrazolium bromide (MTT) assay.

***Results.*** Both adhesives were moderately toxic for HGF cells on the first day of the experiment, but the TiO_2_-containing adhesive produced significantly lower toxicity than the pure adhesive (P<0.05). No significant differences were found in cell viability percentages between the two groups on the other days (P>0.05). There was a significant reduction in cell toxicity with increasing pre-incubation time (P<0.001). L929 cells showed similar toxicity trends, but lower sensitivity to detect cytotoxicity of dental composites.

*** Conclusion.*** The orthodontic adhesive containing TiO_2_ nano-particles indicated comparable or even lower toxicity than its nano-particle-free counterpart, indicating that incorporation of 1 wt% TiO2 nano-particles to the composite structure does not result in additional health hazards compared to that occurring with the pure adhesive.

## Introduction

To date, various techniques have been used to reduce or minimize enamel demineralization in patients with fixed orthodontic appliances. Besides the use of fluoride prophylactic agents, which is usually dependent on patient cooperation, attempts have been made to employ anti-caries adhesives for bonding orthodontic attachments. This may be an efficient way for caries prevention, as the rough surface of remaining adhesive on the tooth surface is more susceptible to plaque accumulation and retention than sound enamel or bracket materials.^1 -3^Furthermore, food accumulation usually occurs around orthodontic brackets and the use of anti-caries adhesives may be effective to counteract the side effects of food accumulation, i.e. plaque formation and white spot lesions. Initially, adhesives or cements that released soluble antimicrobial agents such as chlorhexidine were introduced, but further studies revealed that the anti-caries effect is short-term and is achieved at the expense of degradation in mechanical properties such as reduction in bond strength.^4,5^Another way to obtain an anti-caries composite is by incorporating nano-particles within the resinous matrix to benefit from their antibacterial properties. It is believed that the antibacterial effect of nano-composites is achieved through decreased bacterial adhesion to the composite surface and not because of releasing antibacterial agents to the liquid media or the oral environment.^6^

Nanotechnology has been used in dentistry to provide materials with enhanced mechanical properties and antibacterial effects. The question of “which nano-material is the most suitable for insertion into bonding systems” has long been posed. A recent study found that experimental composites containing silver nano-particles provided superior antibacterial properties without compromising the shear bond strength.^6^ However, incorporation of silver nano-particles may result in discoloration of the composite matrix and there is some concern regarding their biocompatibility.^7,8^ Copper- and zinc-based nano-particles also produced severe toxic effects in animal studies in vitro.^9-11^ Recently, there has been much attention to the photocatalytic activity of titanium-dioxide (TiO_2_) nano-particles in medical and dental literature. Previous studies have indicated that resins containing TiO_2_ nano-particles exhibit significant antimicrobial effects,^12-14^ which may be useful in prevention of recurrent caries and enamel demineralization. Addition of TiO_2_ nano-particles to dental composites also enhanced mechanical properties, including elastic modulus, microhardness and flexural strength,^15,16^ and provided bond strength values that were equal or even higher than that of the nano-particle-free controls.^13,15^

Biocompatibility of different types of orthodontic adhesives and their ingredients have been investigated in several studies and most indicated cytotoxic effects of different intensity,^17-20^ due to the release of unbound molecules from the structure of cured composites.^17,21^ Incorporation of nano-particles to composite structure may result in additional health hazards because of the specific structural and chemical properties of nanoparticles.^22^ To the best of the authors’ knowledge, there is no study regarding the biocompatibility of composites containing TiO_2_ nano-particles. This investigation, thus, aimed to evaluate the effect of incorporating TiO_2_ nano-particles on cytotoxicity of an orthodontic adhesive (Transbond XT) using culture media of human gingival fibroblasts (HGF) and mouse L929 fibroblasts.

## Materials and Methods

### Preparation of Nano-composite 

Titanium dioxide nano-particles (type P25, Photocatalytic standard) were purchased from Plasma Chem GmbH (Berlin, Germany). According to the supplier, the P25 nano-powder composed of mixed anatase and rutile phases and the particles had an average primary size of 21±5 nm, specific surface of 50±10 m^2^/g and more than 99.5% purity after ignition.

A light-cured orthodontic adhesive (Transbond XT; 3M Unitek, Monrovia, California, USA) was used in this study. The P25 TiO_2_ nano-particles were added to Transbond XT adhesive, at a weight ratio of 1%. A composite mixer (SpeedMixer ^TM ^, FlackTek Inc., Landrum, SC, USA) was used to blend TiO_2_ nano-particles into the adhesive at 3500 rpm for 5 minutes.

###  Sample Preparation (Fabrication)

Conventional Transbond XT adhesive served as the control group and Transbond XT containing TiO_2_ nano-particles was regarded as the experimental group. Ten test specimens were prepared from each adhesive with the use of polyethylene discs measuring 8 mm in diameter. The adhesive material was inserted between the two polyethylene disks and pressed by glass slabs to provide uniform thickness and surface smoothness ( Figure 1A). The excess material was removed with a dental explorer from around the disks and each specimen was photopolymerized for 40 seconds by a quartz tungsten halogen curing apparatus (Coltolux 75; Coltene/Whaledent Inc., Cuyahoga Falls, OH, USA) at a maximum intensity of 1000 mW/cm^2^.


Figure 1.(A) A composite specimen prepared using polyethylene disks and glass slabs; (B) Immersion of composite disks in DMEM.

**A:**
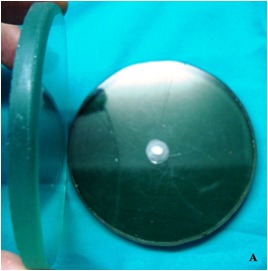

Then, the polyethylene disks were removed from the specimens and the composite thickness was measured with a digital caliper (Guanglu, China) with an accuracy of 0.01 mm. Specimens with a thickness of 0.22±0.05 mm were selected and those with greater or lower thickness were excluded from the experiment. All the samples were prepared by one operator (M.H.).

The specimens were sterilized by exposing them to ultraviolet light for 45 minutes on each side (90 minutes in total) and then were immersed in Dulbecco’s Modified Eagle’s Medium (DMEM; Sigma Chemical Co., St. Louis, Mo, USA) at 37ºC. One specimen of each adhesive was immersed in 1 mL of sterile DMEM (Figure 1B), resulting in a surface area to volume ratio of 1.06 cm^2^/mL. This ratio was within the recommended range of 0.5–6.0 cm^2^/mL suggested by the International Organization for Standardization (1996)^23^ for biological evaluation of medical devices. The material extracts were collected after 1, 3, 5, 7 and 14 days. After each aging interval, the culture medium was replaced with fresh DMEM to simulate the dilution of potentially toxic substances by saliva and fluids in the oral environment. The extracts were sterile-filtered before toxicity experiments on cell cultures.

**B:**
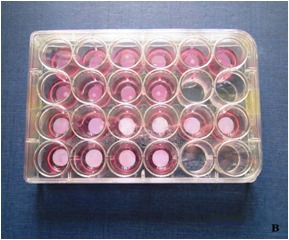


###  Cell Lines and Cell Cultures

Human gingival fibroblasts (HGF) and mouse L929 fibroblasts were used for toxicity assessment; both were purchased from Pasteur Institute, Tehran, Iran. The cells were cultured in DMEM supplemented with 10% (v/v) fetal bovine serum (FBS), 4 mMol of L-gluthamine, 100 units/mL of penicillin, and 100 µg/mL of streptomycin. The cells were kept in an incubator under 5% CO _2 _ at 37ºC.

### Cytotoxicity Assessment 

The toxicity of composite extracts was determined using MTT [3-(4,5-dimethylthiazol-2-yl)-2, 5-diphenyltetrazolium bromide] assay on HGF and L929 cell lines to determine the percentage of viable cells in culture media. In brief, cells (with a density of 8×10^3^ cells per well) were seeded in 96-well plates and incubated for 24 hours at 37°C in a humidified atmosphere of 5% CO_2_, 95% air. After that, the cell culture medium was discarded and 150 µL of adhesive extracts were added. The culture medium by itself was used as the control. After a 24-hour incubation period in a 5% CO_2_ environment, the supernatant was removed and 15 µL (5 mg/mL) of MTT solution (Sigma) was added to the medium in each well and the plates were incubated for 4 hours at 37ºC and 5% CO_2_. Then, the medium with MTT was removed and 100 µL per well of DMSO (dimethyl sulphoxide) solution was added to each well to dissolve the formazan crystals. The optical density (OD) was recorded at 545 nm and the reference OD was 630 nm in an ELISA plate reader. Cell viability was calculated in percentage of control groups according to the following formula:

cell viability (%) = (OD of the test group/ OD of the control group) ×100

Cell viability was then scored according to the following classification:^24^

- more than 90 percent cell viability: non-cytotoxic

- 60–90 percent cell viability: slightly cytotoxic

- 30–59 percent cell viability: moderately cytotoxic

- less than 30 percent cell viability: severely cytotoxic

Ten independent experiments were carried out in triplicate by one operator (AS) for each of the adhesive systems at each aging interval.

###  Statistical Analysis

The normal distribution of the data was confirmed by the Kolmogorov-Smirnov test and the homogeneity of variances with the Levene’s test. Independent-sample t-test was used to delineate any significant difference in cell viability between the two groups at each aging interval. The change in cell viability by increasing pre-incubation period was assessed with one-way analysis of variance (ANOVA). Statistical analysis was performed by Statistical Package for the Social Sciences (SPSS, Version 16.0, Chicago, Illinois, USA) at 95% confidence interval.

## Results

The cytotoxicity results of the two groups in HGF and L929 cell cultures are presented in (Tables 1 and 2, respectively. Considering HGF cells, both adhesives showed moderate toxicity on the first day of the experiment, but cytotoxicity of Transbond XT containing TiO_2_ nano-particles was significantly lower than that of the pure adhesive (P=0.029). Cell survival rate was increased on the third day with no significant differences between the two groups (P>0.05). On days 5 and 7 of the experiment, both adhesives were mildly toxic for HGF cells, and there were no significant differences in cell viability between the study groups either on day 5 or on day 7 (P>0.05). Both adhesives caused more than 100% cell viability on the 14th day.

**Table 1:** Descriptive statistics and the results of Student’s t-test for cell viability (%) comparisons of HGF cells ex-posed to extracts of conventional and TiO_2_-containing adhesives

Groups	Day 1	Day 3	Day 5	Day 7	Day 14
	Mean±SD	Mean±SD	Mean±SD	Mean±SD	Mean±SD
Transbond XT	36±6.53	60.9±7.1	72.6±5.3	83±6.6	102.2±13.4
TiO_2_-containing Transbond XT	43.5±7.6	59.9±5.4	72.8±3.3	88.6±4.4	106.2±16.6
Statistical comparison	P=0.029^*^	P=0.703	P=0.921	P=0.073	P=0.262
^*^statistically significant difference at P<0.05

**Table 2:** Descriptive statistics and the results of Student’s t-test for cell viability (%) comparisons of L929 cells ex-posed to extracts of conventional and TiO_2_-containing adhesives

Groups	Day 1	Day 3	Day 5	Day 7	Day 14
	Mean±SD	Mean±SD	Mean±SD	Mean±SD	Mean±SD
Transbond XT	63.1±5.9	70.5±6.9	78.9±3.9	90.6±6.1	99±10.9
TiO_2_-containing Transbond XT	60±6.3	66±7.5	80.2±3.5	93.5±2.9	99.8±9.3
Statistical comparison	P=0.154	P=0.246	P=0.450	P=0.195	P=0.665

Regarding L929 cell line, both TiO_2_-containing and pure adhesives caused mild decreases in cell survival rates on days 1, 3 and 5 of the experiment, with no significant differences from each other (P>0.05). The extracts of the two composites were essentially not toxic to L929 cells after 7 and 14 days of pre-incubation, and no significant differences were found between them (P>0.05).

**Figure 2.**
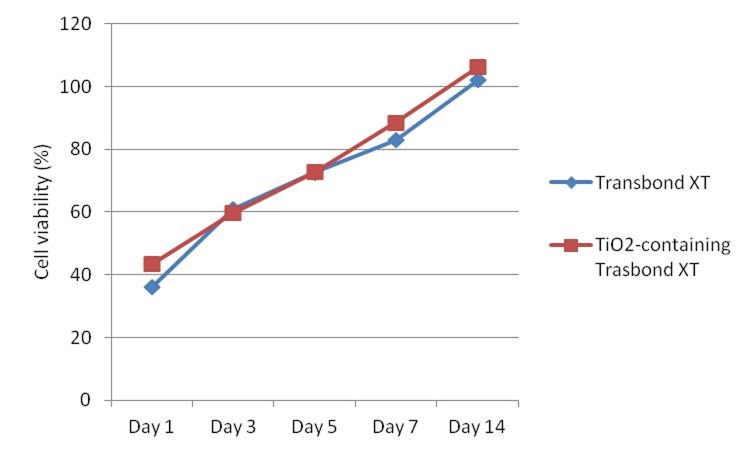
Cell viability of HGF cells exposed to extracts of conventional and TiO_2_-containing adhesives with different pre-incubation periods.

**Figure 3.**
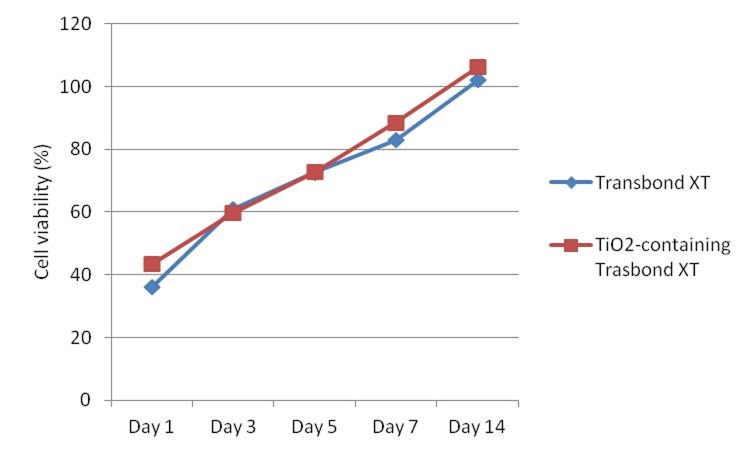
Cell viability of L929 cells exposed to extracts of conventional and TiO_2_-containing adhesives with different pre-incubation periods.

Aging had a significant effect on cell viability (P<0.001), so that the cytotoxicity of both adhesives decreased with increasing pre-incubation periods (Figures 2 and 3). Cell viability trends were similar in both groups and both cell lines during the course of the experiment.

## Discussion

The present study evaluated cytotoxicity of a photo-initiated orthodontic adhesive containing 1 wt% TiO_2_ nano-particles. The concentration of TiO_2_ nano-particles was selected according to a previous study,^13^ which found that incorporation of 1 wt% TiO_2_ nano-particels to Transbond XT adhesive brought about significant antimicrobial properties without decreasing shear bond strength. The adhesive specimens were prepared in the same thickness as that used under brackets to provide data relevant to the clinical conditions.^17,25^ HGF cells were selected because of their abundance in the gingiva where excess bonding material would be frequently observed. The MTT assay used in this study is a simple, sensitive, reliable and commonly used enzymatic assay to determine the cytotoxicity of various medicinal and toxic materials. The test is based on metabolic activity of viable cells to convert a yellow, soluble tetrazolium salt (MTT) into formazan dye with purple color.

Various nano-particles are applied in medicine and dentistry. TiO_2_ nano-particles, which are commercially available in different sizes and crystalline structures, are ideal for incorporation into dental materials. They are relatively inexpensive with excellent mechanical properties and desirable color.^15,16^ The anatase phase of TiO_2_ nano-particles shows high photocatalytic activity, including killing bacteria, fungi and viruses. The ultrafine TiO<sub>2</sub> nano-particles used in this study were mixtures of anatase and rutile phases, with anatase being the main crystalline structure.

In the present study, viability of HGF cells decreased considerably after exposure to extracts of TiO_2_-containing and conventional adhesives. Cytotoxicity of the tested nano-composite was significantly lower than that of the pure resin on the first day of the experiment, but no significant difference was found between the two groups on the other days. The relatively high cytotoxicity of Transbond XT adhesive can be explained by considering its ingredients, as a mixture of different resins such as bisphenol A diglycidyl dimethacrylate (Bis-GMA) and triethylene glycol dimethacrylate (TEGDMA) are used in its chemical composition. Malkoc et al^26^ attributed the cytotoxicity of Transbond XT system to the presence of bisphenol A-ethoxylate dimethacrylate (Bis-EMA) in its matrix. It has been indicated that substantial amounts of monomers and short chain polymers remain unbound within the structure of cured bonding resins,^27,28^ and their release has been considered the main cause of cytotoxic reactions to composite resins in short term.^17,21^ Besides, biodegradation of dental composites generates leachable substances that can produce comparable toxic effects as the raw monomers.^21,29^ The findings of this study are consistent with those of Pithon et al,^30^ who found severe cytotoxicity for Transbond XT adhesive at 24 hours, which diminished considerably after 48 hours of photo-polymerization, and was related to the degree of monomer conversion. Janke et al^19^ also reported moderate degree of toxicity for freshly prepared Transbond XT adhesive, and Santos et al^20^ reported that Transbond XT showed the worst biocompatibility in rat subcutaneous tissue among the adhesives tested. The results of this study, however, are in contrast with those of some previous authors^17,26^ who found just mild toxicity for Transbond XT system after 24 to 72 hours of pre-incubation.

Ageing resulted in a significant decrease in cytotoxicity of both adhesives during the course of the experiment, so the extracts were not toxic to L929 or HGF cells after 7 or 14 days of pre-incubation, respectively. These results are consistent with those of previous authors who reported reduction in cytotoxicity of dental composites with increasing pre-incubation periods,^18,19,31-34^ which was almost immeasurable after 7 days^33,34^ due to the reduced release of unbound molecules into the aqueous media.^27,28^

The use of permanent mouse fibroblast (L929) cells is common in cell culture studies because of the ease of amplification and their relatively consistent and well-known behavior, which makes them useful for in vitro toxicity assessment of dental materials.^26^ It is believed that L929 fibroblasts and gingival fibroblasts show similar cell viability levels.^26^ In the present study, cytotoxicity patterns were almost similar in HGF and L929 cell cultures over the whole period of the experiment, but mouse fibroblasts demonstrated lower sensitivity than human gingival fibroblasts to detect cytotoxicity of orthodontic adhesives, and thus they may underestimate the adverse health effects of dental materials.

Cytotoxicity of different nano-particles has been demonstrated in several studies in dose-dependent and time-dependent manners.^9,10,35,36^ Generally, nano-particles are assumed to cause greater toxicity than fine-size particles due to their greater surface area-to-volume ratio.^22,37^ Cytotoxicity of nano-particles is also determined by other physico-chemical factors including size, concentration, chemical composition, and crystalline structure.^22,38,39^ Recently, Lanone et al^10^ compared the toxicity of 24 nano-particles in the same experimental set-up on two pulmonary cell lines (A549 and THP-1) and found that copper- and zinc-based nano-particles were highly toxic, whereas titania showed moderate degree of toxicity. Warheit et al^39^ reported severe pulmonary inflammatory/toxicity effects in rats after intratracheal instillation of ultrafine anatase/rutile TiO_2_nano-particles. However, in the present study, Transbond XT adhesive containing 1 wt% TiO_2_ nano-particles proved to have comparable and even lower toxicity than the pure adhesive, indicating its potential safety for intraoral applications.

Insertion of nano-particles into bonding systems can be regarded as a method of providing antibacterial activity, which prevents plaque adhesion and caries. The adoption of nano-materials, however, has been assumed to be associated with the risk of biologic hazards to the patient as well as to the practitioner and the dental team. Orthodontic adhesives are in close contact with gingival and oral tissues and thus they should be biocompatible to be applicable in clinical conditions. The findings of this study indicated that incorporation of 1 wt% TiO_2_ nano-particles into a conventional orthodontic adhesive does not lead to additional health hazards compared to that occurring with the pure resin. However, regular cautious measures should be taken when using nano-composites as well as their conventional counterparts, including exact removal of additional composite around bracket bases, especially in gingival and interproximal areas and prevention of dermal contact with resinous materials. It should be emphasized that in vitro assays cannot replicate the complex oral environment. Further studies are warranted to elucidate the in vivo biologic effects of nano-composites including cytotoxic and genotoxic damages to tissues and organs in long-term exposure.

## Conclusion

1. The toxicity of TiO_2_-containing adhesive on HGF cells was significantly lower than that of the conventional light-cured system on day 1, but no significant differences were found between the two groups on the other days.
2. Mouse L929 fibroblasts showed lower sensitivity than HGF cells to detect toxicity of the tested adhesives, but cell viability trends were similar in both cell lines.
3. Aging caused a significant decrease in cytotoxicity of both adhesives in the two media.
4. The Transbond XT adhesive containing TiO_2_ nano-particles proved to have comparable and even better biocompatibility than the pure adhesive, supporting its use as a preventive strategy to reduce the risk of demineralization around fixed orthodontic attachments.

##  Acknowledgments

The authors would like to thank the Vice Chancellor for Research of Mashhad University of Medical Sciences for the financial support of this project (grant no 900072). The results described in this paper were taken from an MSc student thesis (thesis no 457).
